# Supplementary material for: Genotype Prevalence of Lactose Deficiency, Vitamin D Deficiency, and the Vitamin D Receptor in a Chilean Inflammatory Bowel Disease Cohort: Insights from an Observational Study
Source: Int J Mol Sci. 2023 Oct 3;24(19):14866. doi: 10.3390/ijms241914866 (PMC10573577; doi:10.3390/ijms241914866)
Supplement: Supplementary file 1 [file ijms-24-14866-s001.zip › ijms-2612415-supplementary.pdf]

https://www.ebi.ac.uk/gwas/efotraits/EFO_0003762 Accesed 17 July 2023

Variant and risk allele	P-value	P-value annotation	RAF	OR	Beta	CI
rs12785878-?	2 x 10-27	-	0.23	-	-	-
rs2282679-?	2 x 10-109	-	0.29	-	-	-
rs10741657-?	3 x 10-20	-	0.40	-	-	-
rs7129781-C	4 x 10-33	-	0.071	-	0.189 unit inc	[0.16-0.22]
rs4944958-A	9 x 10-143	-	0.203	-	0.259 unit inc	[0.24-0.28]
rs964184-G	3 x 10-14	-	0.129	-	0.095 unit inc	[0.071-0.119]
rs10859995-1	3 x 10-33	-	0.42	-	0.105 unit de	[0.087-0.123]
rs1532085-A	1 x 10-8	-	0.383	-	0.05 unit incr	[0.032-0.068]
rs1800588-T	2 x 10-10	-	0.212	-	0.066 unit inc	[0.046-0.086]
rs55791371-(	1 x 10-9	-	0.121	-	0.081 unit de	[0.056-0.106]
rs10426201-(	8 x 10-20	-	0.169	-	0.106 unit de	[0.082-0.13]
rs3750297-A	3 x 10-10	-	0.283	-	0.059 unit inc	[0.041-0.077]
rs12123821-1	6 x 10-26	-	0.047	-	0.227 unit de	[0.18-0.27]
rs4845491-C	7 x 10-10	-	0.054	-	0.119 unit de	[0.082-0.156]
rs8123293-G	3 x 10-8	-	0.113	-	0.076 unit de	[0.049-0.103]
rs17217119-(	5 x 10-16	-	0.193	-	0.086 unit inc	[0.064-0.108]
rs3755322-G	6 x 10-12	-	0.083	-	0.104 unit inc	[0.075-0.133]
rs6600893-C	5 x 10-15	-	0.452	-	0.067 unit inc	[0.049-0.085]
rs2282679-G	1 x 10-200	-	0.274	-	0.429 unit inc	[0.41-0.45]
rs2205262-C	5 x 10-11	-	0.429	-	0.057 unit de	[0.039-0.075]
rs11723621-?	2 x 10-24	-	NR	-	1.28 ng/mL ir	[-18.7708-21.
rs11023332-?	3 x 10-11	-	NR	-	0.77 ng/mL ir	[-12.2444-13.
rs7041-?	2 x 10-9	-	NR	-	0.79 ng/mL ir	[-11.0288-12.
rs12803256-?	4 x 10-8	-	NR	-	0.65 ng/mL ir	[-10.13-11.43
rs3831470-?	1 x 10-6	-	NR	-	0.58 ng/mL ir	[-9.024-10.18
rs78359207-?	2 x 10-6	-	NR	-	0.59 ng/mL ir	[-8.7788-9.95
rs55715230-?	3 x 10-6	-	NR	-	0.66 ng/mL ir	[-8.5324-9.85
rs306141-?	3 x 10-6	-	NR	-	0.6 ng/mL inc	[-8.5336-9.73
rs17382663-?	5 x 10-6	-	NR	-	0.91 ng/mL d	[-8.008-9.828
rs58788626-?	7 x 10-6	-	NR	-	0.84 ng/mL d	[-7.98-9.66]

Mapped gene	Reported trai	Trait(s)	Background trait(s)	Study accessi	Location
NADSYN1	Vitamin D ins	vitamin D def	-	GCST000697	11:71456403
GC	Vitamin D ins	vitamin D def	-	GCST000697	4:71742666
CYP2R1, CAL(	Vitamin D ins	vitamin D def	-	GCST000697	11:14893332
CYP2R1	Vitamin D ins	vitamin D def	-	GCST90020[illegible]	11:14890871
NADSYN1	Vitamin D ins	vitamin D def	-	GCST90020[illegible]	11:71457027
ZPR1	Vitamin D ins	vitamin D def	-	GCST90020[illegible]	11:116778201
HAL	Vitamin D ins	vitamin D def	-	GCST90020[illegible]	12:95981904
ALDH1A2	Vitamin D ins	vitamin D def	-	GCST90020[illegible]	15:58391167
LIPC, ALDH1A	Vitamin D ins	vitamin D def	-	GCST90020[illegible]	15:58431476
SMARCA4	Vitamin D ins	vitamin D def	-	GCST90020[illegible]	19:11077477
SULT2A1	Vitamin D ins	vitamin D def	-	GCST90020[illegible]	19:47881492
PADI1	Vitamin D ins	vitamin D def	-	GCST90020[illegible]	1:17233181
FLG-AS1	Vitamin D ins	vitamin D def	-	GCST90020[illegible]	1:152206676
LCE6A, SMCP	Vitamin D ins	vitamin D def	-	GCST90020[illegible]	1:152877093
CYP24A1, BC[illegible]	Vitamin D ins	vitamin D def	-	GCST90020[illegible]	20:54102016
BCAS1, CYP2[illegible]	Vitamin D ins	vitamin D def	-	GCST90020[illegible]	20:54126051
UGT1A6, UG[illegible]	Vitamin D ins	vitamin D def	-	GCST90020[illegible]	2:233713141
UGT2B7, UG[illegible]	Vitamin D ins	vitamin D def	-	GCST90020[illegible]	4:69113183
GC	Vitamin D ins	vitamin D def	-	GCST90020[illegible]	4:71742666
LINC00536	Vitamin D ins	vitamin D def	-	GCST90020[illegible]	8:115999659
GC	Vitamin D ins	vitamin D def	-	GCST90101[illegible]	4:71749645
PDE3B	Vitamin D ins	vitamin D def	-	GCST90101[illegible]	11:14762564
GC	Vitamin D ins	vitamin D def	-	GCST90101[illegible]	4:71752617
ACTE1P	Vitamin D ins	vitamin D def	-	GCST90101[illegible]	11:71421822
NADSYN1	Vitamin D ins	vitamin D def	-	GCST90101[illegible]	11:71454899
NPFFR2	Vitamin D ins	vitamin D def	-	GCST90101[illegible]	4:72032699
PACRG	Vitamin D ins	vitamin D def	-	GCST90101[illegible]	6:162756538
LINC01824, L[illegible]	Vitamin D ins	vitamin D def	-	GCST90101[illegible]	2:6458749
PVT1	Vitamin D ins	vitamin D def	-	GCST90101[illegible]	8:128090786
MAP3K9-DT,	Vitamin D ins	vitamin D def	-	GCST90101[illegible]	14:70865550

rs2228570

id	rs10741657	rs12785878	rs2282679	rs4988235	
1	GG	TT	AA	CC	
100	AG	TT	AA	CC	
101	AG	GT	AA	TC	
103	GG	TT	AA	CC	
104	GG	TT	AA	TC	
106	GG	GT	AA	CC	
107	AA	GG	AA	CC	1
109	GG	TT	CA	CC	
11	AG	TT	AA	TC	
111	AG	TT	CC	CC	
112	GG	GT	AA	TC	
114	GG	TT	AA	CC	
115	AG	GT	CA	TC	
116	GG	GT	CA	TC	
117	GG	GG	CA	CC	2
118	AG	GT	AA	CC	
119	GG	TT	AA	TC	
121	AA	GT	CA	CC	
122	GG	GT	CA	CC	
123	AG	TT	AA	TC	
124	GG	TT	AA	TC	
125	GG	GG	AA	CC	3
127	AG	TT	CA	CC	
128	GG	GG	AA	TC	3
129	AG	GT	CA	CC	
13	GG	GT	AA	CC	
130	AG	GG	AA	TC	
131	AG	TT	AA	TC	
133	GG	TT	CA	CC	
134	AG	TT	AA	TC	
135	AG	GT	AA	CC	
136	GG	GT	CA	TC	
137	AG	TT	AA	CC	
138	AA	GG	AA	TC	
139	GG	TT	AA	TT	
14	AG	GG	AA	TC	
140	AA	GT	AA	TC	
141	AG	GT	CA	CC	
142	AG	GT	CA	CC	
143	GG	GT	CA	CC	
144	AG	TT	AA	TC	
145	AA	TT	AA	CC	

146	AG	TT	AA	CC	
147	GG	TT	CA	TC	
148	AG	TT	CA	TC	
149	AG	GT	AA	CC	
150	GG	TT	CC	TC	
151	AG	TT	AA	CC	
152	AG	TT	AA	CC	
153	AA	GT	AA	CC	
154	GG	GT	CA	CC	
156	GG	TT	CA	TC	
158	GG	GT	CA	TC	
159	AG	GT	AA	TC	
16	AG	GT	CA	CC	
160	AG	GT	AA	CC	
161	GG	GT	CA	CC	
162	AG	GG	CA	CC	4
163	GG	GT	AA	TC	
164	GG	GT	CC	CC	
165	AG	GG	AA	CC	5
166	AG	GG	CA	CC	6
167	GG	GG	AA	TC	4
169	GG	TT	AA	CC	
17	GG	GG	AA	CC	7
170	AA	GT	AA	CC	
171	AG	GT	CA	TC	
172	AA	GT	CA	CC	
173	GG	TT	CA	CC	
174	AG	GT	AA	CC	
175	AA	GT	AA	CC	
177	GG	TT	CC	CC	
179	AG	GT	AA	TC	
18	AG	TT	CC	TC	
180	AG	TT	AA	TC	
181	GG	GT	CA	TC	
183	AG	GT	CA	TC	
184	GG	TT	AA	CC	
185	AA	GT	CA	CC	
186	AG	GG	AA	TC	
187	AG	TT	AA	CC	
188	AG	GT	AA	CC	
189	GG	GT	AA	CC	
19	GG	GG	AA	CC	8
190	AG	TT	CA	TC	

191	AA	GT	CA	CC	
192	AA	GG	AA	TC	
193	GG	GT	CA	TC	
194	AG	TT	AA	TC	
195	AG	GT	CA	CC	
196	GG	TT	CA	CC	
197	AG	GT	AA	TC	
198	AA	GT	CA	CC	
199	AG	GG	AA	TC	
2	AG	TT	AA	TT	
20	AG	GG	AA	CC	9
200	AA	TT	AA	CC	
201	AG	TT	CA	CC	
202	GG	GT	CA	CC	
203	GG	TT	AA	TC	
204	GG	GG	AA	TC	7
205	AA	GT	CC	CC	
206	AG	GT	CA	TC	
207	GG	GG	AA	CC	10
208	GG	TT	AA	TC	
209	GG	TT	AA	CC	
21	GG	GG	AA	CC	11
210	GG	GT	AA	TC	
211	AG	GG	AA	TT	
212	GG	GG	AA	TC	10
213	AG	TT	AA	TC	
215	AG	GT	CA	CC	
22	GG	GT	AA	CC	
23	AG	GT	AA	CC	
24	AG	GT	AA	CC	
25	GG	GT	AA	TC	
26	GG	GG	AA	TC	11
27	GG	GT	CA	CC	
28	AA	GT	AA	TC	
29	AG	GT	AA	TC	
30	GG	GG	AA	CC	12
31	GG	GT	CA	CC	
32	AA	TT	AA	CC	
33	AA	TT	AA	CC	
34	AG	GG	CA	CC	13
35	GG	TT	CA	TT	
36	GG	GG	AA	CC	14
37	AG	TT	CC	CC	

38	AG	GT	CA	TC	
39	AG	TT	CA	CC	
4	AA	GT	CA	CC	
40	GG	GT	AA	CC	
41	AG	GG	CA	CC	15
42	AA	TT	AA	CC	
43	AG	GT	AA	TC	
44	AG	GT	AA	CC	
45	AG	GT	AA	TC	
46	GG	TT	AA	TC	
47	AG	TT	CA	TC	
48	GG	GT	CA	CC	
49	AG	GG	AA	CC	16
5	AG	TT	AA	CC	
50	AG	GG	CA	CC	17
51	AG	GT	CA	TC	
52	AG	GT	CA	CC	
53	AG	TT	CC	TC	
54	AG	GT	AA	TC	
55	AG	TT	CA	CC	
56	GG	GT	AA	CC	
57	AG	GG	AA	CC	18
58	GG	GT	AA	CC	
59	GG	TT	CC	CC	
6	AG	TT	AA	CC	
60	GG	TT	AA	TC	
61	AG	GG	CA	CC	19
62	GG	GT	CA	CC	
63	GG	TT	CA	TC	
64	AG	TT	CA	CC	
65	GG	GT	AA	CC	
66	AG	GT	AA	TC	
67	GG	TT	CA	TC	
68	GG	TT	AA	CC	
69	AG	GT	AA	CC	
7	GG	GT	AA	CC	
70	GG	GG	AA	TT	14
71	GG	GT	CA	CC	
74	AG	GG	AA	CC	20
75	AG	GT	AA	CC	
76	GG	GG	AA	CC	21
78	GG	GT	AA	TC	
79	GG	GT	AA	CC	

8	AG	TT	CA	CC	
80	GG	TT	AA	CC	
81	AG	GG	CA	CC	22
82	GG	GT	CA	CC	
83	GG	GT	AA	TC	
85	AG	GT	AA	CC	
86	GG	GT	AA	TC	
87	AG	GG	AA	TC	
88	GG	GT	AA	TC	
89	AG	GT	AA	TC	
9	GG	TT	AA	TC	
90	GG	GT	AA	CC	
91	GG	TT	CA	CC	
92	GG	GT	AA	CC	
93	GG	GT	AA	CC	
94	AG	TT	AA	CC	
95	GG	GT	CA	CC	
96	GG	GT	CC	TC	
97	GG	TT	CA	CC	
98	AG	GG	AA	CC	23
99	AG	GT	CA	CC	

rs10741657-GG and rs4988235-GG

patient	rs11568820	rs1544410	rs7975232	seq.rs2228570
1	GG	GG	AC	AA
100	GG	GG	CC	AA
101	AG	AG	AC	AA
103	GG	AA	AA	AA
104	AG	AG	AC	AA
106	GG	GG	CC	AA
107	GG	AG	AA	AA
109	GG	AG	AC	AA
11	GG	GG	AA	AA
111	GG	GG	CC	AA
112	GG	GG	CC	AA
114	GG	GG	CC	AA
115	GG	AG	AA	AA
116	GG	AG	AC	AA
117	GG	AG	AC	AA
118	GG	AG	AC	AA
119	GG	GG	CC	AA
121	GG	GG	CC	AA
122	GG	AG	AC	AA
123	GG	AG	AA	AA
124	GG	GG	CC	AA
125	GG	GG	AA	AA
127	GG	AG	AC	AA
128	GG	GG	AA	AA
129	AG	GG	AC	AA
13	GG	GG	CC	AA
130	GG	AG	AA	AA
131	GG	GG	CC	AA
133	GG	GG	CC	AA
134	GG	AG	AC	AA
135	AG	AA	AA	AA
136	GG	GG	AC	AA
137	GG	AG	AC	AA
138	AG	GG	CC	AA
139	GG	GG	CC	AA
14	GG	AG	AC	AA
140	GG	GG	CC	AA
141	AG	GG	CC	AA
142	GG	GG	AC	AA
143	GG	GG	CC	AA
144	GG	GG	CC	AA
145	GG	GG	AC	AA

146	GG	AG	AC	AA
147	GG	AG	AC	AA
148	AG	AG	AA	AA
149	GG	GG	CC	AA
150	AG	AG	AA	AA
151	GG	AA	AA	AA
152	GG	AG	AC	AA
153	GG	AG	AC	AA
154	GG	AG	AA	AA
156	GG	GG	CC	AA
158	AA	GG	AC	AA
159	GG	AG	AC	AA
16	GG	GG	CC	AA
160	AG	AG	AA	AA
161	GG	AG	AA	AA
162	AG	AG	AA	AA
163	GG	GG	CC	AA
164	AG	AG	AC	AA
165	GG	GG	CC	AA
166	GG	AG	AA	AA
167	AG	AA	AA	AA
169	GG	GG	AC	AA
17	AG	GG	CC	AA
170	GG	AG	AC	AA
171	GG	AG	AC	AA
172	GG	GG	CC	AA
173	GG	GG	CC	AA
174	GG	GG	AC	AA
175	GG	GG	CC	AA
177	GG	AG	AA	AA
179	GG	AG	AC	AA
18	GG	GG	CC	AA
180	AG	GG	AC	AA
181	AG	GG	AC	AA
183	AA	AG	AC	AA
184	GG	AG	AC	AA
185	GG	GG	AC	AA
186	AG	AA	AA	AA
187	GG	GG	AC	AA
188	AG	GG	AA	AA
189	GG	GG	AA	AA
19	GG	GG	AC	AA
190	AG	GG	CC	AA

191	AG	AG	AC	AA
192	GG	GG	CC	AA
193	GG	GG	CC	AA
194	GG	AG	AA	AA
195	GG	AG	AC	AA
196	GG	AG	AA	AA
197	AG	GG	CC	AA
198	AG	GG	CC	AA
199	AG	GG	CC	AA
2	AG	AG	AA	AA
20	GG	AG	AC	AA
200	GG	GG	CC	AA
201	GG	GG	AC	AA
202	GG	GG	AC	AA
203	AG	AG	AA	AA
204	GG	GG	AC	AA
205	GG	GG	CC	AA
206	GG	AG	AC	AA
207	GG	AG	AC	AA
208	GG	GG	CC	AA
209	GG	GG	AC	AA
21	GG	GG	CC	AA
210	GG	AG	AC	AA
211	GG	GG	CC	AA
212	AG	GG	CC	AA
213	GG	AG	AC	AA
215	AA	AG	AC	AA
22	AG	AA	AA	AA
23	GG	GG	AA	AA
24	GG	AG	AC	AA
25	GG	AG	AC	AA
26	GG	GG	CC	AA
27	GG	GG	AC	AA
28	GG	AG	AC	AA
29	GG	GG	CC	AA
30	AG	GG	AC	AA
31	GG	AG	AC	AA
32	AA	AA	AA	AA
33	AA	AA	AA	AA
34	GG	GG	CC	AA
35	GG	GG	AC	AA
36	GG	GG	CC	AA
37	GG	AG	AC	AA

38	GG	GG	AC	AA
39	GG	GG	CC	AA
4	AG	GG	CC	AA
40	GG	GG	AC	AA
41	GG	GG	AC	AA
42	GG	AG	AC	AA
43	GG	AG	AC	AA
44	GG	GG	AA	AA
45	GG	GG	CC	AA
46	GG	GG	AA	AA
47	GG	AG	AA	AA
48	AG	GG	AA	AA
49	GG	GG	AC	AA
5	GG	GG	AC	AA
50	GG	GG	CC	AA
51	GG	GG	CC	AA
52	GG	AA	AA	AA
53	GG	GG	AC	AA
54	GG	GG	AC	AA
55	AG	GG	AC	AA
56	GG	GG	CC	AA
57	GG	AG	AA	AA
58	GG	AG	AC	AA
59	AG	AG	AA	AA
6	AG	AG	AC	AA
60	GG	AG	AC	AA
61	GG	AG	AA	AA
62	GG	AG	AA	AA
63	GG	GG	AA	AA
64	GG	AG	AC	AA
65	GG	GG	CC	AA
66	GG	GG	AC	AA
67	AG	AG	AC	AA
68	GG	GG	AC	AA
69	GG	AG	AA	AA
7	GG	GG	CC	AA
70	GG	GG	AC	AA
71	GG	AG	AC	AA
74	AG	AG	AC	AA
75	GG	GG	AC	AA
76	GG	GG	AC	AA
78	AG	AG	AC	AA
79	AG	AG	AC	AA

8	GG	GG	AC	AA
80	GG	GG	CC	AA
81	GG	GG	CC	AA
82	GG	GG	CC	AA
83	GG	GG	CC	AA
85	GG	GG	AC	AA
86	GG	GG	AA	AA
87	GG	GG	CC	AA
88	GG	GG	AA	AA
89	GG	GG	AC	AA
9	AG	AG	AC	AA
90	GG	AG	AC	AA
91	GG	AG	AC	AA
92	GG	GG	AC	AA
93	AG	AG	AC	AA
94	GG	GG	CC	AA
95	GG	AG	AA	AA
96	AG	GG	AC	AA
97	GG	GG	CC	AA
98	GG	GG	AA	AA
99	GG	AA	AA	AA

		z				
						TT/TC/CC
2	rs4988235	136608646	ALL(NP)	T	C	5/70/117
	Risk Variant	Chr	Gene	Phenotype	Genotype	
	rs4988235	2			TT	TC
				Lactose Mala	5(2.6%)	70(36.4%)
	rs228679	4			CC	CA
				Deficiency Vit	10(5.2%)	65(33.8%)
	rs12785878	11			GG	GT
				Deficiency Vit	37(19.3%)	88(45.8%)
	rs10741657	11			AA	AG
				Deficiency Vit	20(10.4%)	86(44.8%)

European lactase genotype determines lactase phe

Table 3 General characteristics, self-reported gastrointestinal symp
C>T$_{-13910}$ single nucleotide polymorphism genotype and allele frequ
individuals

	Hispanics n=216
Women (%)	46
Age (years)	50±12
N° self-reporting lactose intolerance	44 (20.4%)
N° reporting diarrhoea	15 (7%)
N° reporting bloating	77 (35.6%)
LCT-13910 CC genotype	123 (56.9%)
LCT-13910 CT genotype	90 (41.7%)
LCT-13910 TT genotype	3 (1.4%)
C allele frequency	77.7%
T allele frequency	22.3%

	192	216	
Risk Variant	IBD Chilean	Hispanic Chile	Amerindian*
rs4988235	C=0.79	C=0.77	C=0.94
	T=0.21	T=0.22	T=0.6

0.3646	0.3299	0.1903
	Allele Frequency	
CC	C=0.79	
117(61%)	T=0.21	
AA	C=0.22	
117(61%)	A=0.88	
TT	G=0.42	
67(34.9%)	T=0.58	
GG	G=0.67	
86(44.8%)	A=0.33	

G|G: 0.631
A|A: 0.063
A|G: 0.305

Risk Variant	Chr
rs4988235	
	2
rs228679	
	4
rs12785878	
	11
rs10741657	
	11

notype in Chilean population

otoms related to dairy ingestion and lactase (LCT)
uencies in a population of Hispanic and Amerindian

	Amerindians n=43	p Value
	65	0.02
	54±15	NS
	19 (44.1%)	0.001
	16 (37.2%)	<0.001
	14 (32.5%)	NS
	38 (88.3%)	<0.001
	5 (11.7%)	<0.001
	0	NS
	94.2%	<0.001
	5.8%	<0.001

11	rs12785878
4	rs2282679
11	rs10741657

AMR*	EUR*	EAS*	AFR*
C=0.78	C=0.49	C=1	C=0.97
A=0.22	T=0.51	T=0	A=0.3

	C	T
IBD Chilean	0.79	0.21
Hispanic Chile	0.77	0.22
Amerindian C	0.94	0.6
AMR	0.78	0.22

EUR	0.49	0.51
EAS	1	0
AFR	0.97	0.03

(219)
(22)
(106)

Aym	Map	Eur	Afr
0,10781973	0,29773621	0,57101775	0,0234263
0,00001	0,00001	0,021482	0,00001
0,263526	0,854842	0,991303	0,200194

Gene	Allele Freque	Phenotype	Genotype			
	C=0.79		TT	TC	CC	C=0.79
	T=0.21	Lactose Mala	5(2.6%)	70(36.4%)	117(61%)	T=0.21
	C=0.22		CC	CA	AA	C=0.22
	A=0.88	Deficiency Vit	10(5.2%)	65(33.8%)	117(61%)	A=0.88
	G=0.42		GG	GT	TT	G=0.42
	T=0.58	Deficiency Vit	37(19.3%)	88(45.8%)	67(34.9%)	T=0.58
	G=0.67		AA	AG	GG	G=0.67
	A=0.33	Deficiency Vit	20(10.4%)	86(44.8%)	86(44.8%)	A=0.33

71167449	ALL(NP)	G	T	37/88/67
72608383	ALL(NP)	C	A	10/65/117
14914878	ALL(NP)	A	G	20/86/86

Risk Variant	Allele Freque	Genotype			All
		Chile-IBD			All
rs4988235	C=0.79	TT	TC	CC	C: 0.839 (4200)
	T=0.21	5(2.6%)	70(36.4%)	117(61%)	T: 0.161 (808)
rs2282679	C=0.22	CC	CA	AA	ALL
	A=0.88	10(5.2%)	65(33.8%)	117(61%)	A: 0.798 (3996)
					C: 0.202 (1012)
					All
rs12785878	G=0.42	GG	GT	TT	G: 0.646 (3235)
	T=0.58	37(19.3%)	88(45.8%)	67(34.9%)	T: 0.354 (1773)
					All
rs10741657	G=0.67	AA	AG	GG	**A: 0.308 (1541)**
	A=0.33	20(10.4%)	86(44.8%)	86(44.8%)	**G: 0.692 (3467)**
VDDR and iBD					**All**
rs2228570		AA	AG	GG	**A: 0.328 (1645)**
	A=1	192	0	0	**G: 0.672 (3363)**
	G=0				

			European
TT	TC	CC	C: 0.492 (495)
197(0.979%)	414(0.165%)	1893(0.756%)	T: 0.508 (511)
			European
CC: 0.047 (118)	**CA: 0.310 (776)**	**AA: 0.643 (1610)**	A: 0.753 (758)
**118**	776	1610	C: 0.247 (248)
			European
G|G: 0.465 (1164)	G|T: 0.362 (907)	**T|T: 0.173 (433)**	G: 0.299 (301)
1164	907	433	T: 0.701 (705)
			European
**A|A: 0.097 (244)**	**A|G: 0.421 (1053**	**G|G: 0.482 (1207)**	A: 0.381 (383)
244	1053	1207	G: 0.619 (623)
**A|A: 0.125 (312)**	**A|G: 0.408 (1021**	**G|G: 0.468 (1171)**	
312	**1021**	1171	

<table>
<tr><td></td><td></td><td></td><td>American</td><td></td></tr>
<tr><td>TT</td><td>TC</td><td>CC</td><td>G: 0.784 (544)</td><td>TT</td></tr>
<tr><td>162(0.322%)</td><td>187(0.372)</td><td>154(0.306)</td><td>A: 0.216 (150)</td><td>22(0.063)</td></tr>
<tr><td></td><td></td><td></td><td>American</td><td></td></tr>
<tr><td>CC: 0.054 (27)</td><td>CA: 0.386 (194)</td><td>AA: 0.561 (282)</td><td>T: 0.791 (549)</td><td>CC: 0.032 (11</td></tr>
<tr><td>27</td><td>194</td><td>282</td><td>G: 0.209 (145)</td><td>11</td></tr>
<tr><td></td><td></td><td></td><td>American</td><td></td></tr>
<tr><td>G|G: 0.097 (49)</td><td>G|T: 0.404 (203)</td><td>T|T: 0.499 (251)</td><td>G: 0.550 (382)</td><td>G|G: 0.320 (1</td></tr>
<tr><td>49</td><td>203</td><td>251</td><td>T: 0.450 (312)</td><td>111</td></tr>
<tr><td></td><td></td><td></td><td>American</td><td></td></tr>
<tr><td>A|A: 0.153 (77)</td><td>A|G: 0.455 (229)</td><td>G|G: 0.392 (197)</td><td>A: 0.284 (197)</td><td>A|A: 0.075 (2</td></tr>
<tr><td>77</td><td>229</td><td>197</td><td>G: 0.716 (497)</td><td>26</td></tr>
</table>

		Asian			
TC	GG	G: 1.000 (100	TT	TC	CC
106(0.305)	219(0.631)				504
		Asian			
CA: 0.354 (12	AA: 0.614 (213)	T: 0.739 (745	G|G: 0.067 (3	**G|T: 0.387 (**	T|T: 0.546 (27
123	213	G: 0.261 (263	34	195	275
		Asian			
G|T: 0.461 (1	**T|T: 0.219 (76)**	**G: 0.620 (62**	**G|G: 0.377 (**	**G|T: 0.486 (**	**T|T: 0.137 (**
160	76	**T: 0.380 (38**	190	245	69
A|G: 0.418 (1	**G|G: 0.507 (176)**	A: 0.319 (322	A|A: 0.097 (4	A|G: 0.444 (2	**G|G: 0.458**
145	176	G: 0.681 (686	49	224	231

75)

(69)

(231)

	TT	TC	CC	P Value (Chi square)
Chilean-IBD	5	70	117	0.4
Chilean-Hispanic*	3	90	126	Reference
Amerindians*	0	5	38	0.02

117/192 (60%)	Chilean-IBD
126/219(58%)	Chilean-Hispanic*
38/43(88%)	Amerindians*

	rs4988235 Genotypes			
	TT	TC	CC	P Value Chi square
Chilean-IBD	5	70	117	Reference
All	197	414	1893	7.74x10-12
European	162	187	154	<2.2x10-16
American	22	106	219	0.08
East Asian	0	0	504	<2.2x10-16

	rs2282679			
	CC	CA	AA	P Value Chi square
Chilean-IBD	10	65	117	Reference
All	118	776	1610	0.645
European	27	194	282	0.494
American	11	123	213	0.493
East Asian	34	195	275	0.302

	rs12785878			
	GG	GT	TT	P Value Chisquare
Chilean-IBD	37	88	67	Reference
All	1164	907	433	1.32x10-14
European	49	203	251	0.0001
American	111	160	76	0.0005
East Asian	190	245	69	7.24x10-11

	rs10741657			
	AA	AG	GG	
Chilean-IBD	20	86	86	Reference
All	244	1053	1207	0.659
European	77	229	197	0.175
American	26	145	176	0.302
East Asian	49	224	231	0.949

	rs2228570			
	AA	AG	GG	
Chilean-IBD	192	0	0	Reference
All	312	1021	1171	<2.2 x10-16
European	80	220	203	<2.2 x10-16
American	83	169	95	<2.2 x10-16
East Asian	92	237	175	<2.2 x10-16

	rs1544410			
	AA	AG	GG	
Chilean-IBD	10	73	109	Reference
All	281	920	1303	0.03
European	89	228	186	3.47x10-7
American	27	124	196	0.5
East Asian	0	55	439	<2.2 x10-16

	rs11568820			
	GG	AG	AA	
Chilean-IBD	148	39	5	Reference
All	941	838	725	<2.2 x10-16
European	303	171	29	0.0001
American	235	97	15	0.06
East Asian	189	231	84	<2.2 x10-16

	rs7975232			
	CC	AC	AA	
Chilean-IBD	58	88	46	Reference
All	657	734	1113	3.32x10-8
European	115	170	21	2.84x10-5
American	116	75	156	3.54x10-9
East Asian	261	50	193	<2.2 x10-16

GG	
37/192	19%
1164/2504	46%
49/503	9%
111/347	32%
190/504	38%

rs12785878	Chilean-IBD (N=192)	All (N=2504)	OR
TT	67(34.9)	433(17.3%)	Reference
GT	88(45.8%)	907(36.2%)	1.59
GG	37(19.3%)	1164(46.5%)	4.86
**rs12785878**	**Chilean-IBD (N=192)**	**European (N=503)**	**OR**
TT	67(34.9)	251(49.9%)	Reference
GT	88(45.8%)	203(40.4%)	0.61
GG	37(19.3%)	49 (9.7%)	0.35
**rs12785878**	**Chilean-IBD (N=192)**	**American (N=347)**	**OR**
TT	67(34.9)	76(21.9%)	Reference
GT	88(45.8%)	160(46.1%)	1.60
GG	37(19.3%)	111(32%)	2.64
**rs12785878**	**Chilean-IBD (N=192)**	**East Asian (N=504)**	**OR**
TT	67(34.9)	69(13.7%)	Reference
GT	88(45.8%)	245(48.6%)	2.70

rs1544410	Chilean-IBD (N=192)	All (N=2504)	OR
GG	109 (56.8%)	1303 (52%)	Reference
AG	73(38%)	920(36.8%)	1.05
AA	10(5.2%)	281(11.2%)	2.35
**rs1544410**	**Chilean-IBD (N=192)**	**European (N=503)**	**OR**
GG	109 (56.8%)	186(37%)	Reference
AG	73(38%)	228(45.3%)	1.83
AA	10(5.2%)	89(17.7%)	5.21
	**Chilean-IBD**		

46/88/58

rs1544410	(N=192)	American	OR
GG	109 (56.8%)	196(56.2%)	Reference
AG	73(38%)	124(35.7%)	0.94
AA	10(5.2%)	27(7.8%)	1.50
**rs1544410**	**Chilean-IBD (N=192)**	**East Asian**	**OR**
GG	109 (56.8%)	439(87.1%)	
AG	73(38%)	65(12.9%)	0.22

rs11568820	Chilean-IBD (N=192)	All (N=2504)	OR
GG	148(77.1%)	941(37.6%)	Reference
GA	39(20.3%)	838(33.5%)	3.37
AA	5(2.6%)	725(29%)	22.80
**rs11568820**	**Chilean-IBD (N=192)**	**European (N=503)**	**OR**
GG	148(77.1%)	303(60.2%)	Reference
GA	39(20.3%)	171(34%)	2.14
AA	5(2.6%)	29(5.8%)	2.83
**rs11568820**	**Chilean-IBD (N=192)**	**East Asian (N=504)**	**OR**
GG	148(77.1%)	189(37.5%)	Reference
GA	39(20.3%)	231(45.8%)	4.63
AA	5(2.6%)	84(16.7%)	13.15
**rs11568820**	**Chilean-IBD (N=192)**	**American (N=347)**	**OR**
GG	148(77.1%)	235(67.7%)	Reference
GA	39(20.3%)	97(28%)	1.56
AA	5(2.6%)	15(4.3%)	1.88

rs7975232	Chilean-IBD (N=192)	All (N=2504)	OR
CC	58	657(26.2%)	Reference
AA	46	734(29.3%)	1.40
AC	88	1113(44.4%)	1.11
**rs7975232**	**Chilean-IBD (N=192)**	**European (N=503)**	**OR**

CC	58	115(22.9%))	Reference
AA	46	170(33.8%)	1.86
AC	88	218(43.3%)	1.24
**rs7975232**	**Chilean-IBD (N=192)**	**American (N=347)**	**OR**
CC	58	116(33.4%)	Reference
AA	46	75(21.6%)	0.81
AC	88	156(45%)	0.88
**rs7975232**	**Chilean-IBD (N=192)**	**Asian (N=504)**	**OR**
CC	58	261(51.8%)	Reference
AA	46	50(9.9%)	0.24

CI	P value
Reference	1.32x10-14
1.13-2.23	
3.21-7.38	

CI	P value
Reference	0.0001
0.42-0.88	
0.21-0.58	

CI	P value
Reference	0.0005
1.05-2.43	
1.61-4.32	

rs12785878

CI	P value
Reference	7.24x10-11
1.78-4.09	

CI	P value
Reference	0.03
0.77-1.43	
1.21-4.59	

CI	P Value
Reference	3.47x10-7
1.28-2.60	
2.60-10.45	

CI	P Value
Reference	0.5
0.65-1.37	
0.70-3.21	

CI	P Value
	< 2.2e-16
0.14-0.32	

CI	P.value
Reference	<2.2 x10-16
2.34-4.86	
9.30-55.89	

CI	P.Value
Reference	0.0001
1.43-3.19	
1.07-7.46	

CI	P.Value
Reference	<2.2 x10-16
3.10-6.93	
5.20-33.26	

CI	P Value
Reference	0.06
1.02-2.39	
0.67-5.30	

CI	P.Value
Reference	0.24
0.94-2.10	
0.79-1.57	

CI	P.Value

Reference	0.02
1.18-2.93	
0.83-1.86	
**CI**	**P.Value**
Reference	0.69
0.50-1.32	
0.58-1.33	
**CI**	**Pvalue**
Reference	1.56x10-8
0.14-0.39	

Subsequently, pres- ence of 5 VDR SNPs was investigated, namely FokI (rs2228570), BsmI (rs1544410), [illegible]

12	seq-rs2228570		48272895	ALL(NP)	0	A	0/0/192
12	rs1544410		48239835	ALL(NP)	A	G	10/73/109
	rs17879735	no					
	rs731236	no					
	rs757343	no					
12	rs11568820		48302545	ALL(NP)	A	G	5/39/148
12	rs7975232		48238837	ALL(NP)	A	C	46/88/58
	rs7109294	no					
	rs1089634[illegible]	no					
	rs732594	no					
	rs2980	no			rs2228570,rs1544410,rs11568820, and rs[illegible]		
	rs2980	no					

Chromosome 12

		rs1544410	rs7975232	rs2228570	rs11568820
12	rs1544410	1	0.36		0.05
	rs7975232	0.36	1		0.02
	rs2228570			1	
	rs11568820	0.05	0.02		1

Chromosome 11

	rs10741657	rs12785878
rs10741657	1	0.01
rs12785878	0.01	1

ApaI (rs17879735), TaqI (rs731236), and Tru9I (rs757343). Polymorphisms were identified with th

0.0	0.0	1.0
0.3802	0.3671	0.6981
0.2031	0.2226	0.2018
0.4583	0.498	0.3099

s7975232.

12 seq-rs222857 48272895

e restriction di- gest of PCR products.

ALL(NP)	0 A	0/0/192

patient	rs11568820	rs1544410	rs7975232	seq.rs2228570	rs10741657	rs12785878
1	GG	GG	AC	AA	GG	TT
103	GG	AA	AA	AA	GG	TT
106	GG	GG	CC	AA	GG	GT
109	GG	AG	AC	AA	GG	TT
114	GG	GG	CC	AA	GG	TT
117	GG	AG	AC	AA	GG	GG
122	GG	AG	AC	AA	GG	GT
125	GG	GG	AA	AA	GG	GG
13	GG	GG	CC	AA	GG	GT
133	GG	GG	CC	AA	GG	TT
143	GG	GG	CC	AA	GG	GT
154	GG	AG	AA	AA	GG	GT
161	GG	AG	AA	AA	GG	GT
164	AG	AG	AC	AA	GG	GT
169	GG	GG	AC	AA	GG	TT
17	AG	GG	CC	AA	GG	GG
173	GG	GG	CC	AA	GG	TT
177	GG	AG	AA	AA	GG	TT
184	GG	AG	AC	AA	GG	TT
189	GG	GG	AA	AA	GG	GT
19	GG	GG	AC	AA	GG	GG
196	GG	AG	AA	AA	GG	TT
202	GG	GG	AC	AA	GG	GT
207	GG	AG	AC	AA	GG	GG
209	GG	GG	AC	AA	GG	TT
21	GG	GG	CC	AA	GG	GG
22	AG	AA	AA	AA	GG	GT
27	GG	GG	AC	AA	GG	GT
30	AG	GG	AC	AA	GG	GG
31	GG	AG	AC	AA	GG	GT
36	GG	GG	CC	AA	GG	GG
40	GG	GG	AC	AA	GG	GT
48	AG	GG	AA	AA	GG	GT
56	GG	GG	CC	AA	GG	GT
58	GG	AG	AC	AA	GG	GT
59	AG	AG	AA	AA	GG	TT
62	GG	AG	AA	AA	GG	GT
65	GG	GG	CC	AA	GG	GT
68	GG	GG	AC	AA	GG	TT
7	GG	GG	CC	AA	GG	GT
71	GG	AG	AC	AA	GG	GT
76	GG	GG	AC	AA	GG	GG

79	AG	AG	AC	AA	GG	GT
80	GG	GG	CC	AA	GG	TT
82	GG	GG	CC	AA	GG	GT
90	GG	AG	AC	AA	GG	GT
91	GG	AG	AC	AA	GG	TT
92	GG	GG	AC	AA	GG	GT
93	AG	AG	AC	AA	GG	GT
95	GG	AG	AA	AA	GG	GT
97	GG	GG	CC	AA	GG	TT

rs2282679	rs4988235	id
AA	CC	1
AA	CC	103
AA	CC	106
CA	CC	109
AA	CC	114
CA	CC	117
CA	CC	122
AA	CC	125
AA	CC	13
CA	CC	133
CA	CC	143
CA	CC	154
CA	CC	161
CC	CC	164
AA	CC	169
AA	CC	17
CA	CC	173
CC	CC	177
AA	CC	184
AA	CC	189
AA	CC	19
CA	CC	196
CA	CC	202
AA	CC	207
AA	CC	209
AA	CC	21
AA	CC	22
CA	CC	27
AA	CC	30
CA	CC	31
AA	CC	36
AA	CC	40
CA	CC	48
AA	CC	56
AA	CC	58
CC	CC	59
CA	CC	62
AA	CC	65
AA	CC	68
AA	CC	7
CA	CC	71
AA	CC	76

AA	CC	79
AA	CC	80
CA	CC	82
AA	CC	90
CA	CC	91
AA	CC	92
AA	CC	93
CA	CC	95
CA	CC	97

rs2228570,rs1544410,rs17879735,rs731236,rs757343 ,rs11568820 ,rs7975232,rs7109294,rs10896349,rs732594, rs2980

rs2228570
rs1544410
rs17879735
rs731236
rs757343
rs11568820
rs7975232
rs7109294
rs10896349
rs732594
rs2980

rs2228570
rs1544410
rs17879735
rs731236
rs757343
rs11568820
rs7975232
rs7109294
rs10896349
rs732594
rs2980
